# Supplementary material for: Thermodynamics of high-pressure ice phases explored with atomistic simulations
Source: Nat Commun. 2022 Aug 10;13:4707. doi: 10.1038/s41467-022-32374-1 (PMC9365810; doi:10.1038/s41467-022-32374-1)
Supplement: Supplementary file 1 — Supplementary Information [file 41467_2022_32374_MOESM1_ESM.pdf]

Supplementary information: Thermodynamics of high-pressure ice phases explored with atomistic simulations

Aleks Reinhardt,[1] Mandy Bethkenhagen,[2] Federica Coppari,[3] Marius Millot,[3] Sebastien Hamel,[3] and Bingqing Cheng[4,*]

[1]*Yusuf Hamied Department of Chemistry, University of Cambridge, Lensfield Road, Cambridge, CB2 1EW, United Kingdom*

[2]*École Normale Supérieure de Lyon, Université Lyon 1, Laboratoire de Géologie de Lyon, CNRS UMR 5276, 69364 Lyon Cedex 07, France*

[3]*Lawrence Livermore National Laboratory, Livermore, California 94550, USA*

[4]*Institute of Science and Technology Austria, Am Campus 1, 3400 Klosterneuburg, Austria*

(Dated: 2022-07-21)

* bingqing.cheng@ist.ac.at

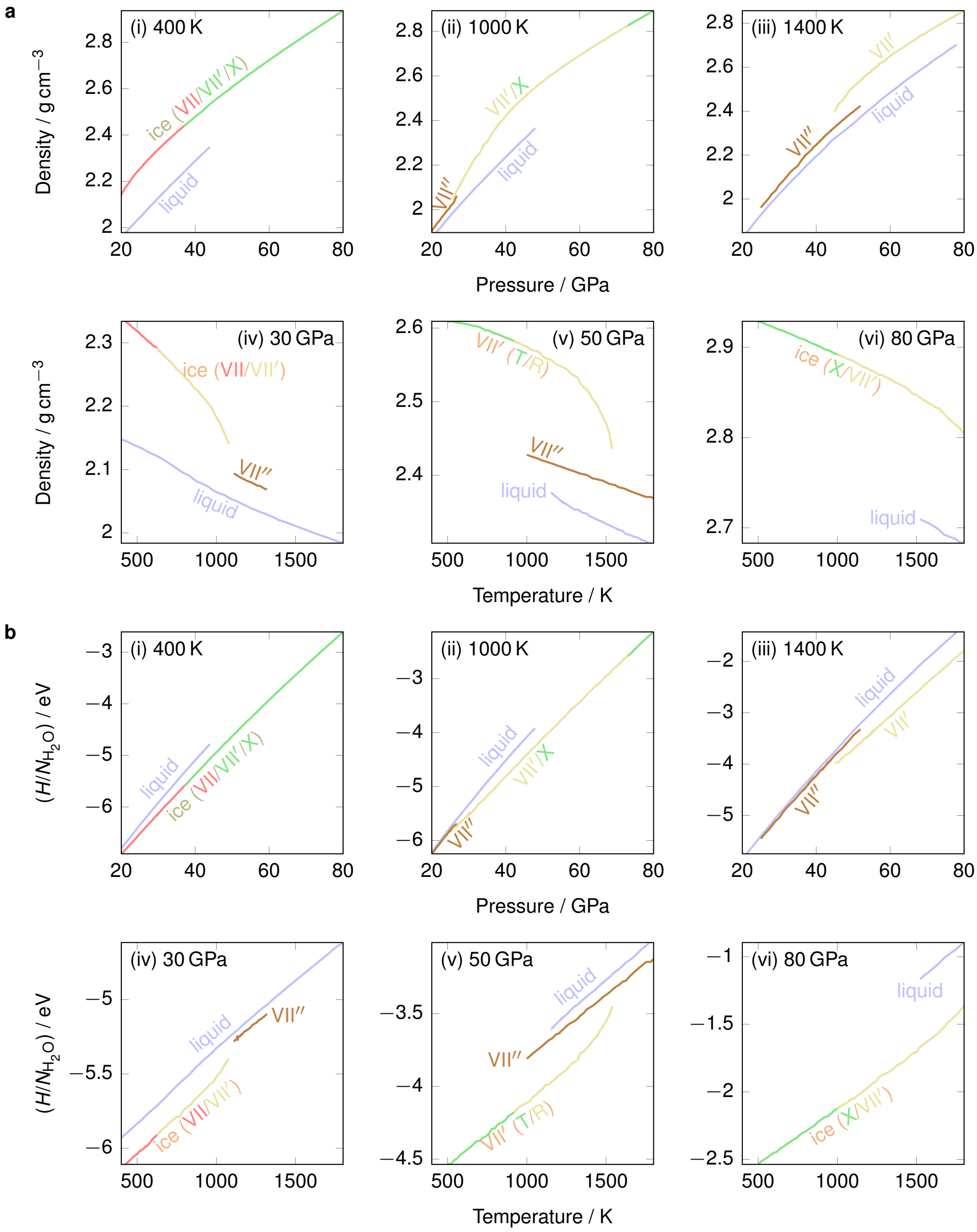

FIG. S1. **Low-pressure phase densities and enthalpies.** **a** Density and **b** enthalpy of the phases indicated along selected isotherms [(i)–(iii)] and isobars [(iv)–(vi)]. Colours are consistent with the main text: red for static ice, green for ice VII (T)/X, yellow for ice VII (R)/X, blue for the liquid and brown for ice VII″.

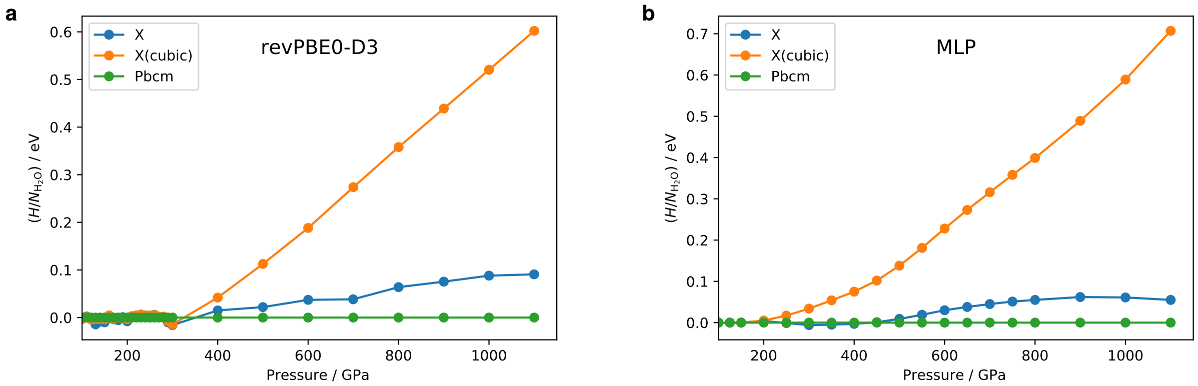

FIG. S2. 0 K enthalpy curves for different ice phases predicted by **a** the revPBE0-D3 DFT functional and **b** the MLP.

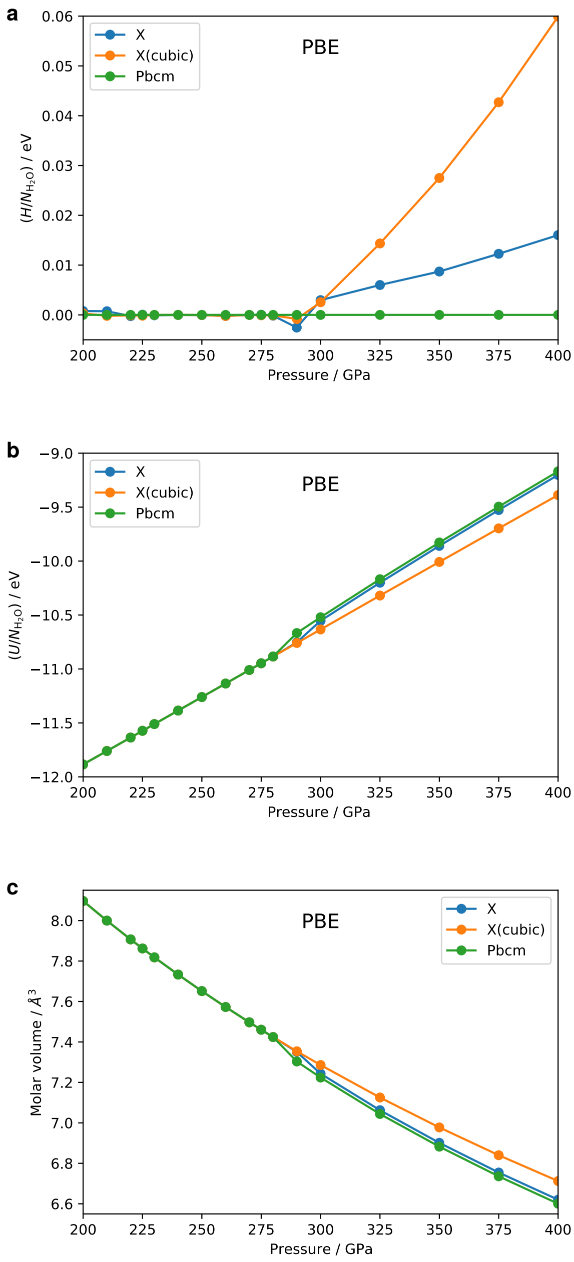

FIG. S3. 0 K **a** enthalpy, **b** potential energy and **c** molar volume for different ice phases predicted by the PBE DFT functional.

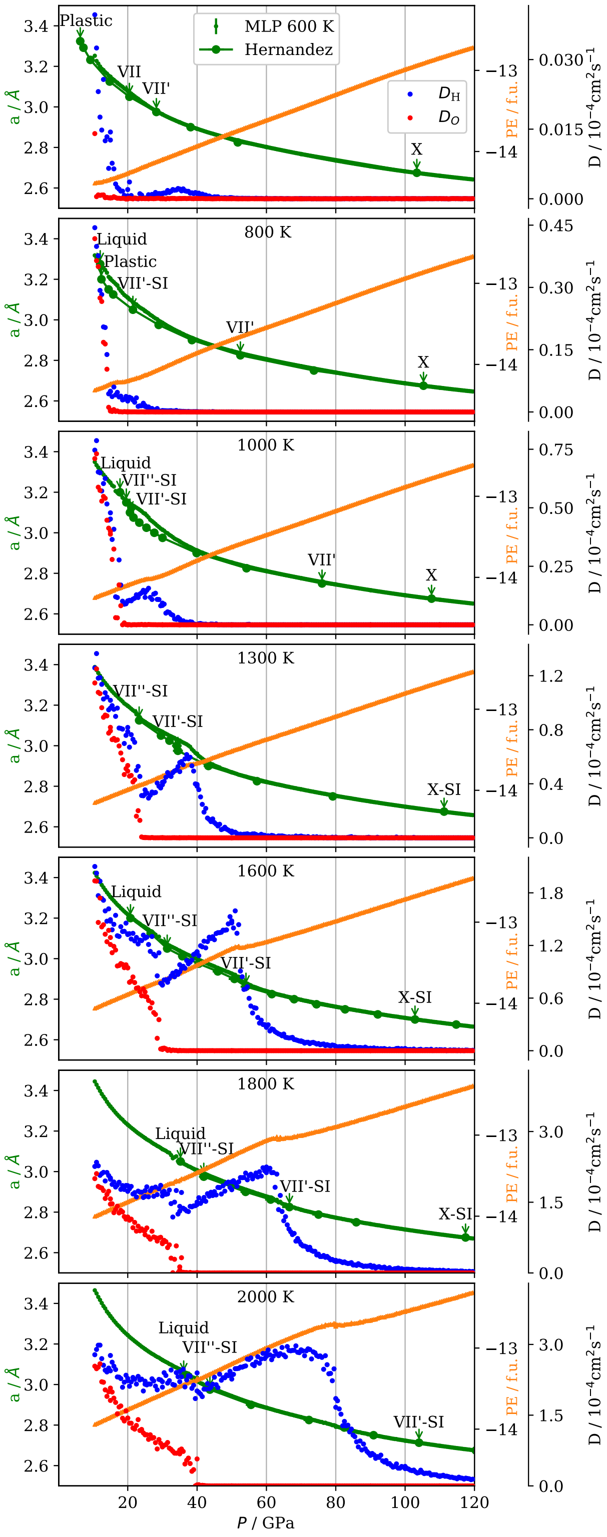

FIG. S4. Equations of states and diffusivity from MD simulations using the MLP and FPMD. The lattice constant data from FPMD (solid green curves) are from NVT simulations of 128 molecules of Hernandez and Caracas [J.-A. Hernandez and R. Caracas, 'Proton dynamics and the phase diagram of dense water ice', J. Chem. Phys. **148**, 214501 (2018)]. The onsets of the pressure where different water phases (e.g. liquid, plastic, VII, VII′, VII′-SI, VII′′-SI, X, X-SI) were observed in FPMD simulations are also reproduced from the same reference, and these pressures are marked by green arrows. The MLP MD data are from NPT simulations using 432 water molecules. The lattice constants are plotted using green dots, and the potential energies per molecule are plotted using orange dots. The error bars are estimated from the standard error of the mean. The red and the blue dots indicate the diffusion coefficient of oxygen and hydrogen atoms, respectively.

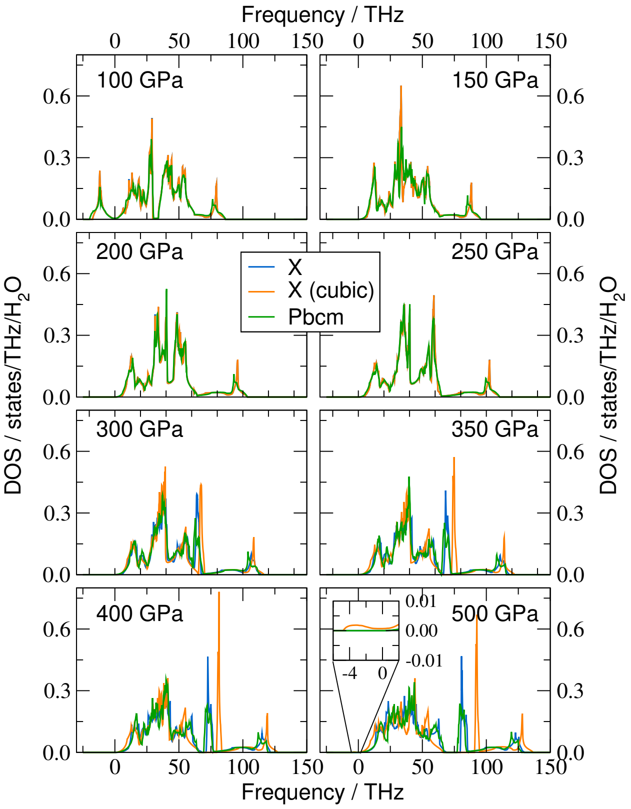

FIG. S5. Phonon density of states (DOS) for the three considered structures.

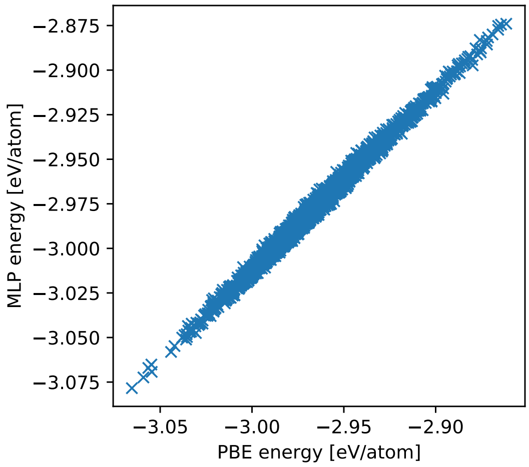

FIG. S6. Comparison between the DFT and the MLP energies for 2,500 snapshots collected from the MLP metadynamics trajectory at 400 GPa and 1000 K.

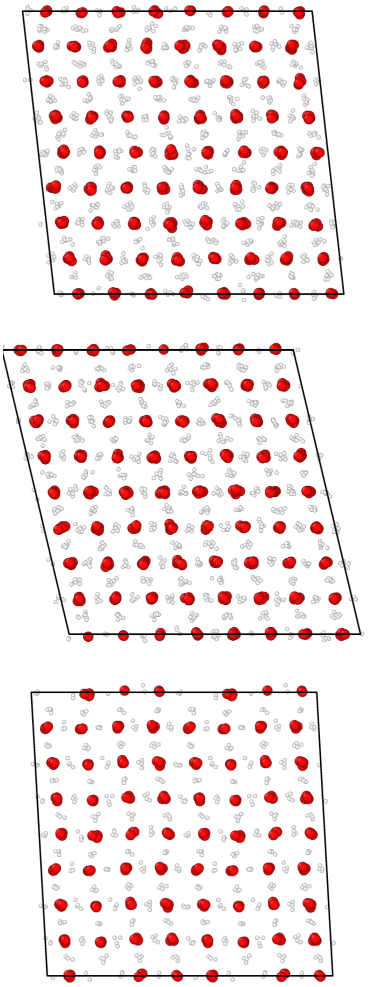

FIG. S7. Structures obtained from metadynamics simulations using 512 water molecules. Top: ice X structure. Middle: structure with stacking faults, similar to SF2 in Fig. 3**d** of the main text. Bottom: another structure with stacking faults, similar to SF1 in Fig.3**d** of the main text.

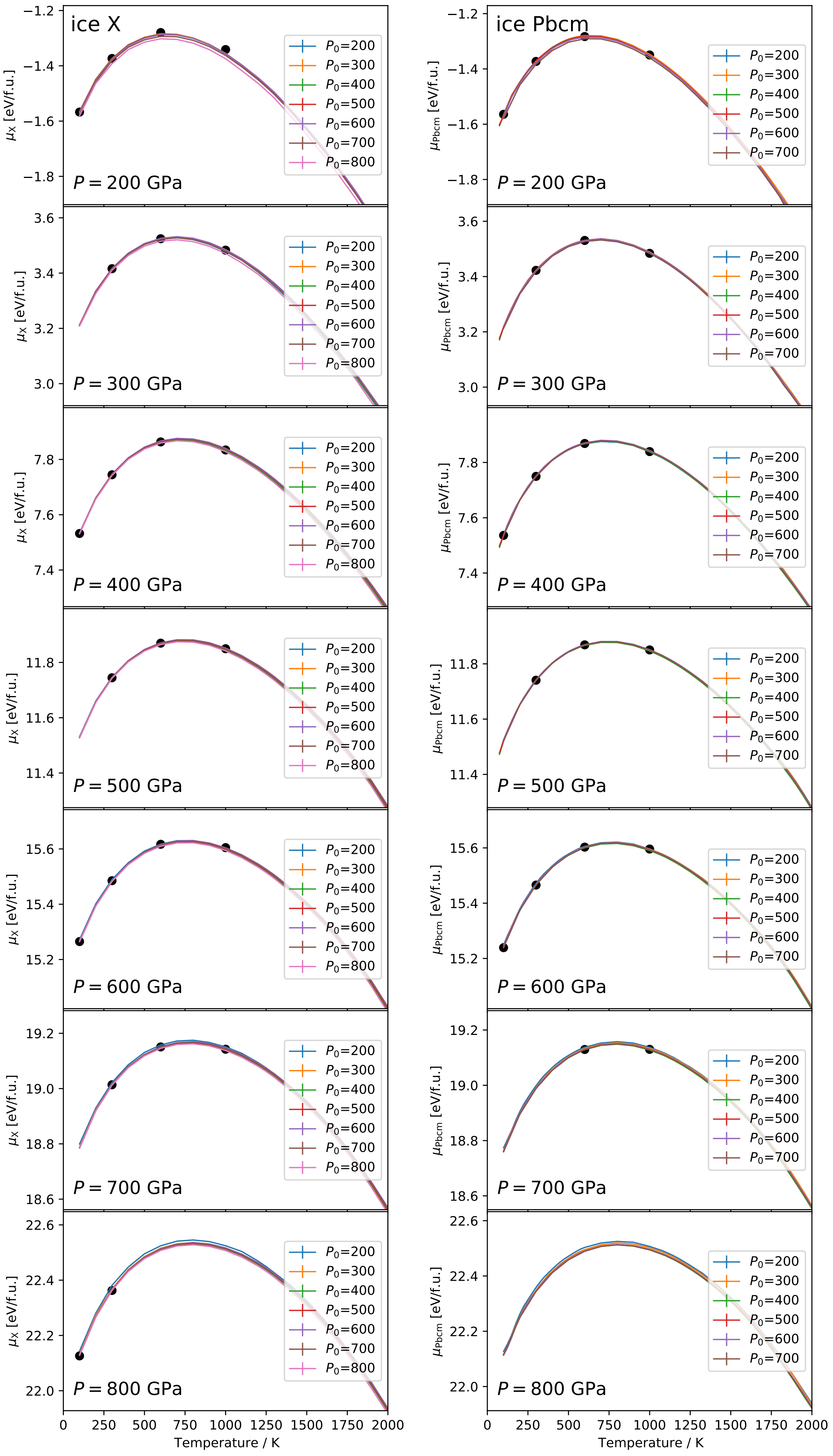

FIG. S8. Chemical potentials of X and P*bcm* computed along different TI routes. The black dots indicate the results obtained from λ-TI at P and the given temperature. The solid lines show the chemical-potential curves obtained from (i) first λ-TI at $T = 300$ K or 600 K at pressure P_0, (ii) integrating along T, and (iii) if $P \neq P_0$, finally integrating along pressure.

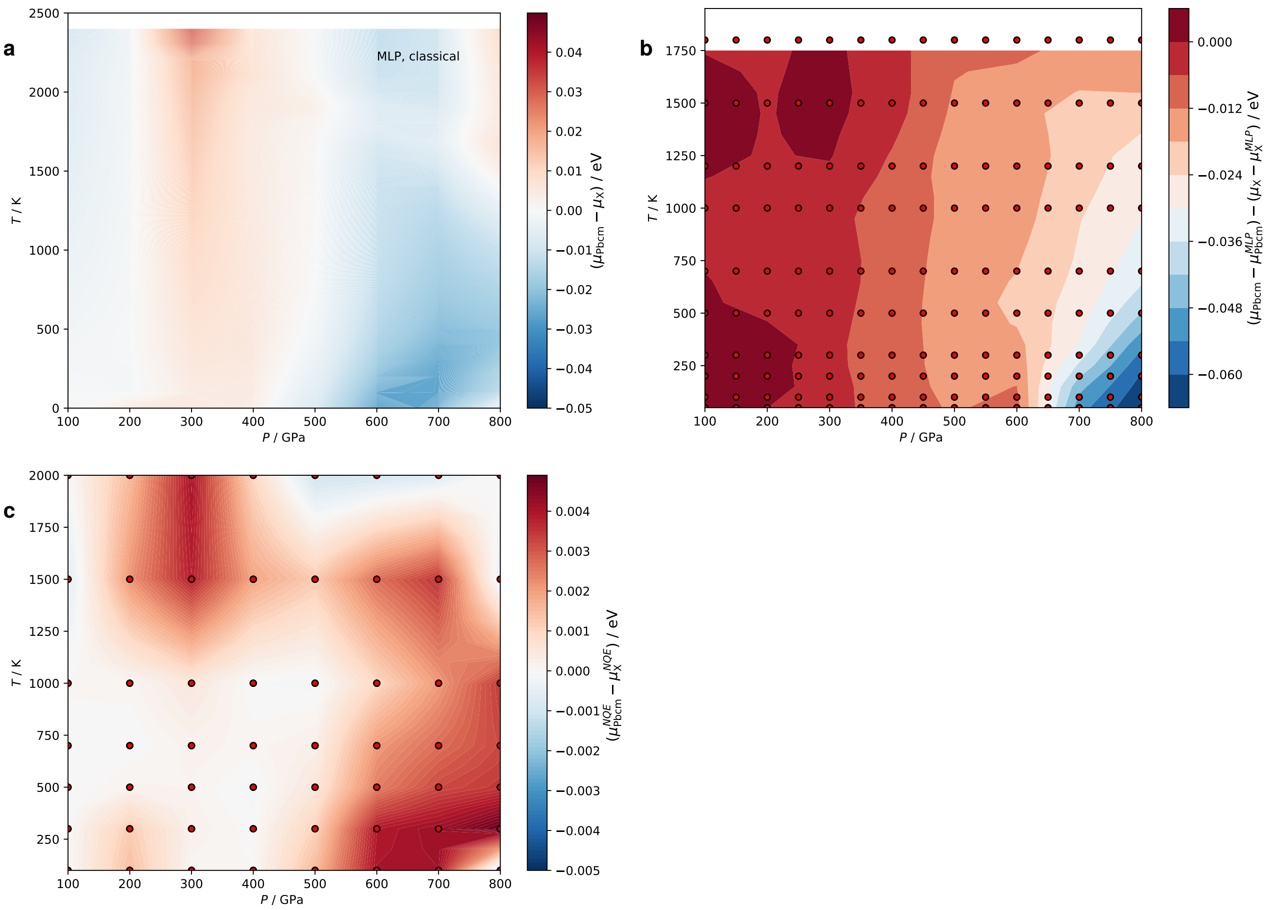

FIG. S9. **a** The chemical-potential difference between P*bcm* and X at the classical nucleus level based on the MLP. **b** The correction term in the chemical-potential difference from MLP to PBE $\mu - \mu^{\text{MLP}}$ computed using the free-energy perturbation method. **c** The contribution of NQEs to the chemical-potential difference. In panels **b** and **c**, red symbols show the conditions at which we explicitly ran simulations to compute the chemical-potential differences, and the colour scales show the interpolated values using these data points.
